# Antibiogram development for Australian residential aged care facilities

**DOI:** 10.1017/ice.2024.120

**Published:** 2024-11

**Authors:** Dipti Khatri, Nazanin Falconer, Sonali Coulter, Leonard C. Gray, David L. Paterson, Christopher Freeman

**Affiliations:** 1 UQ Centre for Health Service Research (CHSR), Faculty of Medicine, The University of Queensland, Woolloongabba, QLD, Australia; 2 School of Pharmacy, The University of Queensland & Princess Alexandra Hospital, Metro South Health, Woolloongabba, QLD, Australia; 3 Pathology Queensland, Microbiology Queensland Public Health and Scientific Services, Herston, QLD, Australia; 4 Faculty of Medicine, The University of Queensland, Metro North Hospital and Health Service, Herston, QLD, Australia; 5 Saw Swee Hock School of Public Health, National University of Singapore, Singapore; 6 School of Pharmacy and Faculty of Medicine, The University of Queensland, QLD, Australia; 7 Metro North Hospital and Health Service, Herston, QLD, Australia

## Abstract

**Objective:**

Knowledge of local antibiotic resistance data, provided by antibiograms (a cumulative summary of in vitro-antimicrobial-susceptibility-test results), can aid prescribing of appropriate empirical antibiotics. This study aimed to explore the feasibility of antibiogram development for residential aged care facilities (RACFs).

**Design:**

Retrospective observational study of culture and sensitivity data.

**Setting:**

Nine RACFs in Queensland, Australia.

**Method:**

Available antimicrobial susceptibility results were collected retrospectively for all residents of recruited RACFs from January 1, 2020, to December 31, 2022. Data were managed and analyzed with WHONET software®, and antibiograms were developed in accordance with the CLSI-M39 guidelines. Antibiogram data beyond the standard 12-months and pooling of data from geographically similar RACFs were explored as options to improve feasibility and validity of the antibiograms.

**Results:**

The most prevalent bacteria in the RACFs were *Escherichia coli* and *Staphylococcus aureus*. Due to the low number of positive cultures (less than 30) for individual RACFs, an annual antibiogram was not feasible. Extending the time-period to three years improved feasibility of antibiograms for *E.coli* in seven RACFs and *S.aureus* in five RACFs. Combining the data from closely located RACFs allowed for sufficient urinary and skin swab isolates to produce annual pooled antibiograms for all three years.

**Conclusion:**

Use of extended time period antibiograms can provide RACF specific urinary and skin/soft tissue resistance data without the necessity of private pathology provider input. However, pooled syndromic antibiograms can be made available on an annual basis, which may be the preferred option.

## Introduction

Prevalence of antimicrobial resistant (AMR) organisms is high in residential aged care facilities (RACFs) and external transmission can worsen health outcomes of the wider community.^1–5^ Empirical antibiotic prescribing may account for up to 85% of prescribing in this setting.^6,7^ Sub-optimal empirical prescribing contributes to inappropriate antibiotic use^8,9^ and may cause harm to patients, delay effective treatment, and promote the use of unnecessarily broad-spectrum antibiotics which can lead to antibiotic resistance.^10–13^ Antimicrobial stewardship (AMS) tools which can guide appropriate empirical prescribing are essential.

Two main sources of information can be used to guide empirical treatment. These include the Therapeutic Guidelines (TG), which guides prescribing in the Australian healthcare settings,^14^ and the cumulative local antibiogram. The TG provide antibiotic treatment recommendations based on national level resistance data sets, which may not consider the local resistance patterns. AMS guidelines advocate for the use of antibiograms and recommend liaising with pathology providers for local microbe epidemiology.^2,15–18^ Site-specific or syndromic antibiograms (eg, specific to urinary tract infections (UTIs)) can increase likelihood of appropriate empiric antibiotic therapy.^19–22^ A recent systematic review suggests that AMS interventions including antibiograms may improve antibiotic use and appropriateness.^23^

Challenges with the development of local antibiograms for RACFs is noted, including low isolate counts.^15,24–29^ Suggested enablers to improve validity of antibiograms (ie, achieving data sufficiency) included increasing the time interval for inclusion of isolates to create biennial or triennial antibiogram. Pooling antibiogram data from RACFs in similar geographical locations could also be considered.

There have been no studies exploring development of antibiograms for Australian RACFs and therefore this study explored the feasibility of antibiogram development in this setting.

## Methods

Approval was obtained from University of Queensland Medical Research Ethics Committee (2022/HE001118) and the clinical governance bodies of each RACF.

This study was a retrospective analysis of available antimicrobial susceptibility results obtained for all residents of recruited RACFs from January 1, 2020, December 31, 2022.

Our approach to the antibiogram development was according to the Clinical and Laboratory Standards Institute (CLSI) M39 Analysis and Presentation of Cumulative Antimicrobial Susceptibility Test Data; Approved Guideline.^15^

Some key recommendations for routine antibiogram development from the CLSI M39 document (Supplementary 1)^15^ are include only verified final diagnostic results rather than surveillance isolates, only include the first isolate of a bacterial species obtained from each patient in the time-period of analysis and only include bacterial species with data for ≥30 isolates. This minimum isolate count is recommended to provide accurate and reasonable calculated susceptibility rates. Reducing the sample size impacts the precision of the reported percent susceptibility with wider the 95% CIs.^15^ The CLSI recommendations are similar to the recommendations provided in Australian hospital antibiogram development guidelines.^30^

CLSI guidelines^15^ suggest that when there are insufficient number of bacterial isolates tested, the “first isolate per 12-month time-period” can be used. In this study, the “first isolate only” and “first isolate per 12-month period” antibiograms were compared for selected larger RACFs to further explore this recommendation. Antibiograms were also analyzed longitudinally to determine whether resistance patterns remain stable enough for data to be combined over two- to three-year period.

### Setting

Ten RACFs (nine metropolitan and one regional), known to the research team, were invited to participate in this study. The RACFs were located in Queensland, Australia, and operated by not-for-profit (religious, charitable, and community) or private organizations. Facilities with 60 or fewer bed numbers were classified as small-sized RACFs, those with 61–100 beds were medium sized, and RACFs with 101 or more beds were considered large facilities.^31^ All RACFs were similar in terms of type of care provided which included a mix of low care, high care, and dementia care residents.

### Data collection

Positive culture results were manually collected for all residents of the RACF for a three-year period—2020 to 2022. Data were collected via online pathology portals when available, through the Medway^32^ (for Queensland Medical Laboratory (QML)), Sonic^33^ (for Sullivan Nicolaides Pathology (SNP)), and Mater Pathology (MP).^34^ All pathology providers use EUCAST^35^ guidelines for interpretation of pathology results. Data were also collected from the residents’ electronic progress notes, and where needed, additional data were sought from residents’ article records.

Details that were collected and documented in an Excel® spreadsheet included resident’s unique identifying number, sex (male/female), date of specimen collection, type of specimen (urine, skin swab, sputum, blood, or other), laboratory used, culture identification number, organism identified, and antibiotic susceptibilities noted as R (resistant), I (intermediate), and S (susceptible).

Data were manually inputted into WHONET 2022®,^36^ a free Windows-based software developed by the WHO Collaborating Centre for Surveillance of Antimicrobial Resistance, based at the Brigham and Women’s Hospital in Boston, USA. It was established for the purpose of managing and analyzing microbiology laboratory data and generates antibiograms and RIS (resistant, intermediate, and susceptible) summary reports.

### Statistical analysis

The methods for statistical comparison of antibiotic susceptibilities among different antibiograms were adapted from CLSI guidelines.^15^

Multiple pathogen–antibiotic pairs (PAPs) were evaluated, including common urinary and skin tissue isolates and their associated cumulative antibiotic susceptibilities. Pathogens included *Escherichia coli* (*E. coli*), *Klebsiella pneumoniae* (*K. pneumoniae*), *Pseudomonas aeruginosa* (*P. aeruginosa*) (urine isolates), *P. aeruginosa* (skin isolates), *Enterococcus faecalis* (*E. faecalis*), and *Staphylococcus aureus* (*S. aureus*). For each bacterial species, several antibiotic susceptibility test results were assessed. The antibiotics were chosen based on the amount of data available and included the oral antibiotics that would be commonly used to treat UTIs and skin/soft tissue infections (SSTI) in aged care settings.^14,37^

For each PAP, cumulative susceptibilities (ie, proportion of susceptible and resistant pathogens) were compared between the different antibiograms using Fisher’s exact test. Differences with a *P* value of equal to or less than .05 were considered statistically significant. All data were managed with Excel® spreadsheets and analyzed using R version 4.3.1®. The 95% confidence intervals for %S were extracted from WHONET®.

## Results

Of the 10 invited RACFs, nine RACFs (eight metropolitan and one regional) participated in this study. Two RACFs were classified as small, one as medium, and six as large (Table 1). Most positive cultures found were from urine samples (n = 751; 70%) followed by skin and soft tissue swabs (n = 300; 28%). Very few positive sputum and blood culture results (n = 36; 3%) were found hence these data were not presented as unlikely to be meaningful. The data entered into WHONET are detailed further in Table 1. The mean age of residents whose samples were included in the antibiograms was 86.3 years and 76.6% of the total samples originated from female residents. The mean number of all positive cultures collected across the nine RACFs for the years 2020, 2021, and 2022 was 42.9 (±14.2), 38.2 (±13), and 39.7 (±16.6), respectively.

**Table 1.** Details of included RACFs, resident demographics, and number of positive cultures included for antibiograms

Facility details			Resident demographics		Positive cultures for inclusion in Antibiograms (entered on WHONET)															
Facility	Bed No.	Size	Mean Age (years)	Female n (%)	Urine isolates				Skin isolates				Other isolates^a^				All isolates			
					2020	2021	2022	Total	2020	2021	2022	Total	2020	2021	2022	Total	2020	2021	2022	Total
**1**	50	Small	84.2	45 (69.2)	16	15	9	40	7	9	8	24	–	1	–	1	23	25	17	**65**
**2**	139	Large	86.7	127 (80.4)	34	38	34	106	20	11	17	48	1	3	–	4	55	52	51	**158**
**3**	109	Large	85.4	60 (82.2)	18	20	19	57	11	1	3	15	–	1	–	1	29	22	22	**73**
**4**	105	Large	88.4	121 (81.8)	29	29	35	93	15	13	19	47	2	5	1	8	46	47	55	**148**
**5**	175	Large	83.9	114 (75.5)	31	31	51	113	10	10	16	36	–	1	1	2	41	42	68	**151**
**6**	60	Small	85.1	59 (73.8)	21	20	25	66	5	6	1	12	1	1	–	2	27	27	26	**80**
**7**	71	Medium	86.4	115 (68.4)	40	39	23	102	22	16	20	58	3	5	–	8	65	60	43	**168**
**8**	154	Large	88	99 (78.6)	42	21	25	88	10	13	12	35	1	1	1	3	53	35	38	**126**
**9**	179	Large	86.8	93 (78.8)	34	24	28	86	9	8	8	25	4	2	1	7	47	34	37	**118**
**Mean** ± **SD**	–	–	86.3	–	29.4 ± 9.3	26.3 ± 8.4	27.7 ± 11.7	83.4 ± 24.3	12.1 ± 5.8	9.7 ± 4.4	11.6 ± 6.9	33.3 ± 15.7	2 ± 1.3	2.2 ± 1.7	1	4 ± 2.9	42.9 ± 14.2	38.2 ± 13	39.7 ± 16.6	120.8 ± 39.3
**Total**	–	–	–	833 (76.6)	265	237	249	751	109	87	104	300	12	20	4	36	386	344	357	**1087**
**Pooled** ^**b**^	971	–	–	–	225	198	226	649	87	71	84	242	9	15	4	28	321	284	314	**919**

a Other isolates include blood, sputum, and eye swab specimens.

b Pooled data exclude Facility 7; resident demographics (mean age and proportion of females) refers to the residents from whom the samples were collected for inclusion in antibiograms.

The participating RACFs use multiple pathology providers, however, majority of the pathology was obtained from QML (89%), followed by SNP (7%).

For urinary isolates*, E. coli* was the most prevalent organism cultured (53%) and for skin swabs, and *S. aureus* was the most prevalent organism (83%). Therefore, the counts of these two organisms were evaluated to explore the feasibility of antibiograms for the individual facilities.

As shown in Table 2, no annual antibiograms had the minimum recommended 30 isolates of either organism. When data were combined for a two-year period, there was insufficient data for *S. aureus*; however, data sufficiency was achieved for *E. coli* isolates in five of the nine RACFs. By combining three years of data, sufficient isolate counts of *S. aureus* were achieved in five RACFs and of *E. coli* in seven of the nine RACFs.

**Table 2.** Description of RACFs and isolate counts for *Staphylococcus aureus* and *Escherichia coli*

Facility details		Number of *Staphylococcus aureus* (Skin/soft tissue specimens)							Number of *Escherichia coli* (Urinary specimens)						
Facility	Bed No.	2020	2021	2022	Biennial^a^	Triennial^a^	Biennial^b^	Triennial^b^	2020	2021	2022	Biennial^a^	Triennial^a^	Biennial^b^	Triennial^b^
**Facility 1**	50	7	9	7	16	23	14	18	10	10	4	14	24	13	21
**Facility 2**	139	16	11	14	25	41	24	35	18	23	24	47	65	38	48
**Facility 3**	109	7	1	3	4	11	4	9	10	10	8	18	28	16	22
**Facility 4**	105	12	11	17	28	40	24	33	11	17	11	28	39	26	33
**Facility 5**	175	9	8	15	23	32	22	30	15	18	27	45	60	40	51
**Facility 6**	60	3	5	1	6	9	6	7	6	12	16	28	34	22	23
**Facility 7**	71	15	12	14	26	41	22	29	17	16	15	31	48	26	38
**Facility 8**	154	8	13	9	22	30	21	27	18	14	19	33	51	29	43
**Facility 9**	179	8	8	7	15	23	15	21	18	13	17	30	48	27	36
**Mean**	–	9.4	8.7	9.7	18.3	27.8	16.9	23.2	13.7	14.8	15.7	30.4	44.1	26.3	35.0
**SD**	–	4.2	3.8	5.6	8.7	12.3	7.6	10.2	4.5	4.2	7.3	10.8	14.0	8.9	11.2
**Pooled (Facility 1**–**6 & 8**–**9)**		70	66	73	–	–	–	–	106	117	126	–	–	–	–

Note. The CLSI M39 guidelines^15^ recommend that only bacterial species with data for ≥30 isolates should be included in the antibiogram. – not relevant; SD, standard deviation.

a First isolate per 12-month period.

b First isolate only; biennial – 2021 and 2022 data combined; Triennial – 2020, 2021; and 2022 data combined.

### Creation of geographically pooled annual antibiogram

To develop the pooled antibiograms, data were combined from facilities 1–6, 8, and 9 due to being co-located within a 30 km radius. The total number of positive isolates increased to 314 for the 2022 pooled data, which is an eightfold increase when compared to mean isolate counts for the individual RACFs. Both *E. coli* and *S. aureus* reached data sufficiency for each of the three 12-month periods when data were combined for RACFs. Pooled regional antibiograms are provided in Supplementary 2. Supplementary 3 includes %S data for individual RACFs and the pooled data with 95% CIs for clinically relevant PAPs with graphical representations for two PAPs.

### Evaluation of first isolate per time-period rule

Biennial and triennial antibiograms for facilities 2 and 7 were selected for comparison analyses due to higher number of isolates tested (Supplementary 4). No statistically significant differences were found between susceptibility rates of chosen pathogen–antibiotic pairs when comparing antibiograms containing first isolate only per two- or three-year time period, versus first isolate per 12-month period.

### Evaluation of resistance patterns over time

Annual antibiograms with pooled data were statistically compared to explore any differences in resistance patterns between data from 2020, 2021, and 2022 (Supplementary 5). There was only one PAP for which a statistically significant difference in susceptibility was noted (*S. aureus*—Clindamycin; *P* = .04) where the data suggest improved susceptibility profile over time.

## Discussion

This study aimed to explore the feasibility of developing RACF-specific antibiograms for potential clinical utility as an AMS tool. Of the nine RACFs included in the study, the recommended 12-month or annual antibiograms were not feasible when CLSI rules of antibiogram development^15^ were considered. For most RACFs, extending the time-period of antibiogram to three years improved feasibility of antibiograms for the most prevalent microorganisms, *E. coli* (seven RACFs) and *S. aureus* (five RACFs). Pooling of data from RACFs in the same geographical location also allowed for development of feasible annual antibiograms.

Our study found similar challenges for antibiogram development in RACFs to other international studies.^24,38^ However, this study is one of the first to investigate potential solutions which could be applied to improve feasibility of antibiogram development (in Australia), as suggested by previous studies.^24–26,28,29^ An American study based in Georgia found that most RACFs had insufficient number of isolates tested to create facility specific antibiogram. They reported that in a given year, 49% of facilities had sufficient *E. coli* isolates tested, but only approximately 10% had sufficient isolates of *K. pneumoniae, P. mirabilis, E. faecalis*, or *P. aeruginosa*.^24^ In a different American study, Taylor and colleagues^38^ also explored feasibility of traditional facility-specific antibiograms for nursing homes (NH) and found that no NHs obtained the minimum of 30 isolates of any single species.

Statistical analyses compared the susceptibility data over a three-year period and assumption of stable resistance patterns was confirmed. Therefore, aggregating data over extended time-periods is a feasible solution in this setting. Although international studies^24,26,28,29^ have advocated for extended time-period antibiograms in RACF or similar settings, the advice surrounding inclusion of “first isolate only” rule was previously unclear. Our findings support CLSI recommendation of the use of first isolate per 12-month period for extended time-period antibiograms as no statistically significant differences were identified when compared to antibiograms which included first isolate only.

The alternative solution to improving antibiogram development feasibility involved pooling RACF data across facilities. This study found this approach as an effective way to achieve data sufficiency allowing for provision of annual antibiograms. The pooled RACFs in this study were located within a 30 km radius. There does not appear to be any guidance on how closely co-located RACFs should be when determining whether the data can be pooled. In this study, all the RACFs were similar in terms of type of care provided. Antibiogram development studies^24,38^ support geography-based antibiograms, such as what was done in this study, as a practical approach to overcoming the challenges of individual RACFs having insufficient data for annual antibiograms. Fridkin and colleagues^24^ justify producing antibiograms at the regional level in Georgia, USA, an area much larger than that which was pooled for in this study. They found that the extent of the differences in the estimated %S for facilities in each region was relatively small, most often <10%, and suggested regional differences were significant in only a small minority of pathogen–antibiotic pairs studied (7 of 23). In comparison to other studies that have evaluated whether the regional (or “pooled”) antibiogram was representative of the individual facility antibiograms,^39,40^ the annual antibiograms in this study had very small number of isolates (less than 30) of the most prevalent pathogens, hence the 95% confidence intervals (CIs) of the %S were wide. This meant a similar analysis using our data would be unreliable.

Taylor and colleagues^38^ found that grouping cultures by region increased the annual mean culture count by 12-fold when compared to the individual RACF annual mean, which was 23.5. The mean culture count was higher in our study (39.3) across the eight RACFs for which data was combined, and pooling data by geographical location increased the isolate count by eightfold. This study finds that the preferred option for provision of resistance data for RACFs are pooled antibiograms based on geographical location. This method allows for creation of annual antibiograms which is the usual recommended time-period. Although extended time-period antibiograms have been found to be feasible, the workload of producing individual antibiograms may be too onerous for RACF nursing staff. Prescribers likely provide clinical care to residents of multiple RACFs, often in the similar location, therefore consulting the same antibiogram may be preferred.

### Strengths and limitations

Although data were collected using multiple search strategies, such as online pathology portals, residents’ online and article progress notes, it is possible that some data could have been missed due to the manual method of data collection. Potential missing data were explored for four RACFs using acquired infection registers and in-depth review of the residents’ progress notes for an eight-month period, which found significant missing pathology results (∼50%) for one of the four RACFs. The primary reason for the missing results was the omission of copying the results to the RACF hence these results were sent to the prescriber only. Overall, there were less than 10% of missing results for the other three RACFs which were mainly due to pathology conducted during hospitalization. When significant pathology results are missed, it would lead to less precise susceptibility and resistance rates. The extraction of the microbiological results directly from private pathology providers would be of benefit to ensure completeness of data.

To create RACF-specific antibiograms with available data, antibiogram development rules were assumed, such as inclusion of diagnostic isolates only. It is possible that residents of RACFs were tested in the absence of signs or symptoms of infection which would mean that any isolates identified from their sample maybe due to asymptomatic colonization. Inclusion of susceptibility results from these isolates may not provide a “true” resistance picture. Infection registers from four of the nine RACFs were obtained to verify whether the samples were sent in presence of signs and symptoms. Our findings indicated that an overwhelming majority of testing was conducted for residents who were symptomatic, such as those with specific clinical signs of UTIs or skin infections. We also acknowledge that our findings are reported for only the most prevalent urinary and skin/soft tissue isolates.

### Future directions

Using geographic locations as the main demarcation to generate a pooled antibiogram can allow private pathology providers or primary health networks to produce simple regional RACF antibiograms to inform nursing home antibiotic stewardship programs. In Australia, the national program for passive surveillance of AMR, known as Australian Passive AMR Surveillance (APAS), collects antimicrobial susceptibility data from pathology providers in public hospitals. Participation in this program is voluntary and not all private pathology providers provide their data to APAS. Multiple private pathology providers service RACFs and should be encouraged to participate in this program so that antibiograms could be made available for RACFs. Further study is warranted to identify any particular challenges faced by pathology providers to submit this data for public health AMR surveillance and antibiogram development purposes.

In addition to the solutions proposed and evidenced in this study, Tolg and colleagues^28^ discuss other potential solutions. Collapsing of data under broader groups, such as gram-negative and gram-positive pathogens, for antibiograms is discussed and suggested to be a good option to increase statistical validity by increasing isolate counts. Limitation of this approach included not providing the prescriber with the full picture and requirement to have some knowledge of intrinsic resistance of certain bacterial species to certain antibiotics. For example, if all urinary isolates were collapsed into gram-positive and gram-negative rows only, the number of isolates tested would increase; however, the gram-negative urinary isolates would include *P. mirabilis*, which is intrinsically resistant to nitrofurantoin. Nitrofurantoin adequately covers other common gram-negative species such as *E. coli* and *K. pneumoniae* hence misinterpretation of the collapsed antibiogram could lead to suboptimal empirical antibiotic choices. This suggestion could be probed further with RACF prescribers. Tolg et al.^28^ also suggest potential use of near-by hospital antibiograms to represent LTCFs’ bacterial susceptibility. Therefore, comparing the RACF antibiograms with other available local resistance data would determine whether this is a suitable option in the Australian landscape.

Most of the study RACFs were located in metropolitan settings. Antibiogram development for regional and rural RACFs should be considered to determine whether findings of this study are representative and generalizable. There is no known reason to suggest that findings cannot be applied to other metropolitan settings in other Australian states.

## Conclusions

Annual antibiograms were not feasible for individual RACFs included in this study due to insufficient isolate counts, ie, meeting the requirement of at least 30 isolates for inclusion in antibiograms. Extending the time-period of the antibiograms to beyond 12 months, specifically to a three-year period, feasibility of urinary and skin swab antibiograms was improved. Pooling antibiogram data from geographically similar RACFs allowed for development of annual antibiograms for most prevalent urinary and skin swab isolates. Although the use of extended time period antibiograms can provide RACF-specific resistance data without the necessity of private pathology provider input, pooled antibiograms can be made available on an annual basis which is the preferred option. Considering a number of residents of RACFs would be admitted to hospital when they have serious infections, the hospital antibiogram could be a relevant proxy when there is insufficient data for a RACF-specific antibiogram. This option would require further research.

## Supporting information

Khatri et al. supplementary material 1 Khatri et al. supplementary material

Khatri et al. supplementary material 2 Khatri et al. supplementary material

Khatri et al. supplementary material 3 Khatri et al. supplementary material

Khatri et al. supplementary material 4 Khatri et al. supplementary material

Khatri et al. supplementary material 5 Khatri et al. supplementary material

## Supplementary material

The supplementary material for this article can be found at https://doi.org/10.1017/ice.2024.120.
