# Supplementary material for: Antibiogram development for Australian residential aged care facilities
Source: Infect Control Hosp Epidemiol. 2024 Sep 26;45(11):1325–31. doi: 10.1017/ice.2024.120 (PMC11663465; doi:10.1017/ice.2024.120)
Supplement: Khatri et al. supplementary material 1 — Khatri et al. supplementary material [file S0899823X2400120Xsup001.docx]

# Supplementary 1

CLSI -M39 Key Recommendations for Routine Antibiogram Development

| Recommendations |
| --- |
| 1. Antibiogram reports should be analysed and presented at least annually |
| 1. Only diagnostic (not surveillance) isolates should be included |
| 1. Only final, verified test results should be included |
| 1. Duplicates should be eliminated by including only the first isolate of a species, patient, and/or analysis period, regardless of specimen course or antimicrobial susceptibility profile |
| 1. Only species with testing data for ≥ 30 isolates should be included |
| 1. Only antimicrobial agents routinely tested against the population of isolates to be analysed should be included, and the %S should be calculated from results reported as well as those that may be suppressed on patient reports for which selective reporting rules have been applied |
| 1. Laboratorians should refrain from including results for supplemental antimicrobial agents selectively tested on resistance isolates only |
| 1. Laboratorians should report %S but exclude the %I (%SDD) in the %S statistic |

Adapted from CLSI-M39 document. Key: %I- percent intermediate; %S-percent susceptible; %SDD -percent susceptible-dose dependent.
