# Supplementary material for: Antibiogram development for Australian residential aged care facilities
Source: Infect Control Hosp Epidemiol. 2024 Sep 26;45(11):1325–31. doi: 10.1017/ice.2024.120 (PMC11663465; doi:10.1017/ice.2024.120)
Supplement: Khatri et al. supplementary material 2 — Khatri et al. supplementary material [file S0899823X2400120Xsup002.docx]

## Supplementary 2

**Urine antibiogram for Pooled RACFs for 2022**

| **Organism** |  | | **Pooled Metropolitan Brisbane RACF antibiogram**  **1 January 2022 – 31 December 2022**  **Cumulative Antimicrobial Susceptibility Test Data**  **– Urine Antibiogram** | | | | | | | | |
| --- | --- | --- | --- | --- | --- | --- | --- | --- | --- | --- | --- |
|  |  | | **PERCENTAGE (%) SUSCEPTIBLE^#^** | | | | | | | | |
|  | **No. isolates** | | **Amoxicillin** | **Amoxicillin/ Clavulanate** | **Cefalexin** | **Nitrofurantoin** | **Trimethoprim** | **Sulfamethoxazole/Trimethoprim** | **Gentamicin** | **Ciprofloxacin** | **Vancomycin** |
| *Escherichia coli* | 126 | % | 58 | 87 | 87 | 98 | 71 | 59 | 96 | 58 | - |
|  |  | n | 126 | 126 | 126 | 125 | 124 | 27* | 113 | 12* |  |
| *Klebsiella pneumoniae* | 24* | % | R | 100 | 96 | 52 | 92 | ^ | 100 | ^ | - |
|  |  | n |  | 24* | 24* | 21* | 24* | 5* | 19* | 1* |  |
| *Pseudomonas aeruginosa* | 14* | % | R | R | R | ^ | R | R | 90 | 82 | - |
|  |  | n |  |  |  | 2* |  |  | 10* | 11* |  |
| *Enterococcus faecalis* | 11* | % | 93 | 100 | R | 93 | R | R | - | - | 100 |
|  |  | n | 14* | 13* |  | 14* |  |  |  |  | 13* |

Key: RACF- Residential aged care facility; ^#^%S for each organism/antibiotic combination generated by including first isolate of organism per 12-month period encountered in a given patient; *Indicates <30 isolates tested and potentially low accuracy of susceptibility rates; -Indicates the antimicrobial agent not tested, or is known to be clinically ineffective; R – intrinsic resistance; ^ results excluded due to insufficient isolates (<10); Antibiotics with light grey shading belong to Access category of Priority Antibacterial List; Antibiotics with dark grey shading belong to Review-Curb category of Priority Antibacterial List, Purple shading – Gram negative pathogens; Blue shading- Gram positive pathogens; Red shading <70% of isolates susceptible; Yellow shading 70-89% of isolates susceptible; Green shading ≥90 of isolates susceptible.

**Skin/Soft tissue antibiogram for Pooled RACFs for 2022**

| **Organism** | **Pooled Metropolitan Brisbane RACF antibiogram**  **1 January 2022 – 31 December 2022**  **Cumulative Antimicrobial Susceptibility Test Data**  **– Skin/Soft tissue Antibiogram** | | | | | | | | | | | | |
| --- | --- | --- | --- | --- | --- | --- | --- | --- | --- | --- | --- | --- | --- |
|  | **PERCENTAGE (%) SUSCEPTIBLE^#^** | | | | | | | | | | | | |
|  | **No. isolates** | | **Amoxicillin/ Clavulanate** | **Penicillin** | **Flucloxacillin** | **Dicloxacillin** | **Cefalexin** | **Sulfamethoxazole/Trimethoprim** | **Erythromycin** | **Tetracycline** | **Ciprofloxacin** | **Vancomycin** | **Clindamycin** |
| *Staphylococcus aureus* | 73 | % | 73 | 16 | 74 | 73 | 73 | 100 | 93 | 99 | 67 | 100 | 93 |
|  |  | n | 73 | 64 | 72 | 63 | 63 | 73 | 72 | 73 | 18* | 21* | 73 |

Key: RACF-Residential aged care facility ; ^#^%S for each organism/antibiotic combination generated by including first isolate of organism per 12-month period encountered in a given patient; *Indicates <30 isolates tested and potentially low accuracy of susceptibility rates; Antibiotics with light grey shading belong to Access category of Priority Antibacterial List; Antibiotics with dark grey shading belong to Review-Curb category of Priority Antibacterial List, Purple shading – Gram negative pathogens; Blue shading- Gram positive pathogens; Red shading <70% of isolates susceptible; Yellow shading 70-89% of isolates susceptible; Green shading ≥90 of isolates susceptible.
