# Supplementary material for: Antibiogram development for Australian residential aged care facilities
Source: Infect Control Hosp Epidemiol. 2024 Sep 26;45(11):1325–31. doi: 10.1017/ice.2024.120 (PMC11663465; doi:10.1017/ice.2024.120)
Supplement: Khatri et al. supplementary material 3 — Khatri et al. supplementary material [file S0899823X2400120Xsup003.docx]

## Supplementary 3

| **Antibiogram** | **E.coli-Trimethoprim** | | | **E.coli-Cefalexin** | | | **E.coli-Nitrofurantoin** | | |
| --- | --- | --- | --- | --- | --- | --- | --- | --- | --- |
|  | %S | 95%CI | n | %S | 95%CI | n | %S | 95%CI | n |
| Facility 1 | 75 | 21.9-98.7 | 4 | 100 | 39.6-100 | 4 | 100 | 39.6-100 | 4 |
| Facility 2 | 79.2 | 57.3-92.1 | 24 | 87.5 | 66.5-96.7 | 24 | 100 | 82.8-100 | 24 |
| Facility 3 | 100 | 59.8-100 | 8 | 100 | 59.8-100 | 8 | 100 | 59.8-100 | 8 |
| Facility 4 | 72.7 | 39.3-92.7 | 11 | 81.8 | 47.8-96.8 | 11 | 100 | 67.9-100 | 11 |
| Facility 5 | 70.4 | 49.7-85.5 | 27 | 88.9 | 69.7-97.1 | 27 | 100 | 84.5-100 | 27 |
| Facility 6 | 26.7 | 8.9-55.2 | 15 | 75 | 47.4-91.7 | 16 | 93.8 | 67.7-99.7 | 16 |
| Facility 7 | 46.7 | 22.3-72.6 | 15 | 73.3 | 44.8-91.1 | 15 | 93.3 | 66.0-99.7 | 15 |
| Facility 8 | 68.4 | 43.5-86.4 | 19 | 84.2 | 59.5-95.8 | 19 | 94.7 | 71.9-99.7 | 19 |
| Facility 9 | 87.5 | 60.4-97.8 | 16 | 88.2 | 62.3-97.9 | 17 | 93.8 | 67.7-99.7 | 16 |
| Pooled (excluding Facility 7) | 71 | 62.0-78.6 | 124 | 86.5 | 79.0-91.7 | 126 | 97.6 | 92.6-99.4 | 125 |
| Pooled-all | 68.3 | 59.8-75.8 | 139 | 85.1 | 77.9-90.3 | 141 | 97.1 | 92.4-99.1 | 140 |

Clinically relevant E.coli-Antibiotics pairs with %Susceptibility (%S) and 95% Confidence Intervals (CI)

Key: *E.coli* – Escherichia coli; n – number of PAPs tested

Clinically relevant S.aureus-Antibiotics pairs with %Susceptibility(%S) and 95% Confidence Intervals (CI)

| **Antibiogram** | **S.aureus-Flucloxacillin** | | | **S.aureus-Cefalexin** | | | **S.aureus-SMX/TMP** | | | **S.aureus-Clindamycin** | | |
| --- | --- | --- | --- | --- | --- | --- | --- | --- | --- | --- | --- | --- |
|  | %S | 95%CI | n | %S | 95%CI | n | %S | 95%CI | n | %S | 95%CI | n |
| Facility 1 | 100 | 56.1-100 | 7 | 100 | 56.1-100 | 7 | 100 | 56.1-100 | 7 | 100 | 56.1-100 | 7 |
| Facility 2 | 78.6 | 48.8-94.3 | 14 | 78.6 | 48.8-94.3 | 14 | 100 | 73.2-100 | 14 | 78.6 | 48.8-94.3 | 14 |
| Facility 3 | 100 | 31.0-100 | 3 | 100 | 31.0-100 | 3 | 100 | 31.0-100 | 3 | 100 | 31.0-100 | 3 |
| Facility 4 | 58.8 | 33.5-80.6 | 17 | 58.8 | 33.5-80.6 | 17 | 100 | 77.1-100 | 17 | 94.1 | 69.2-99.7 | 17 |
| Facility 5 | 93.3 | 66.0-99.7 | 15 | 90 | 54.1-99.5 | 10 | 100 | 74.7-100 | 15 | 100 | 74.7-100 | 15 |
| Facility 6 | 0 | 0.0-94.5 | 1 | 0 | 0.0-94.5 | 1 | 100 | 5.5-100 | 1 | 0 | 0.0-94.5 | 1 |
| Facility 7 | 92.9 | 64.2-99.6 | 14 | 92.9 | 64.2-99.6 | 14 | 100 | 73.2-100 | 14 | 85.7 | 56.2-97.5 | 14 |
| Facility 8 | 66.7 | 30.9-91.0 | 9 | 75 | 35.6-95.5 | 8 | 100 | 62.9-100 | 9 | 100 | 62.9-100 | 9 |
| Facility 9 | 33.3 | 6.0-75.9 | 6 | 0 | 0.0-69.0 | 3 | 100 | 56.1-100 | 9 | 100 | 56.1-100 | 9 |
| Pooled (excluding Facility 7) | 73.6 | 61.7-83.0 | 72 | 73 | 60.1-83.1 | 63 | 100 | 93.8-100 | 73 | 95.2 | 84.1-97.5 | 73 |
| Pooled-all | 76.7 | 66.2-84.9 | 86 | 76.6 | 65.3-85.2 | 77 | 100 | 94.7-100 | 87 | 92 | 83.6-96.4 | 87 |

Key: S.aureus – Staphylococcus aureus; n – number of PAPs tested; SMX/TMP – sulfamethoxazole/trimethoprim


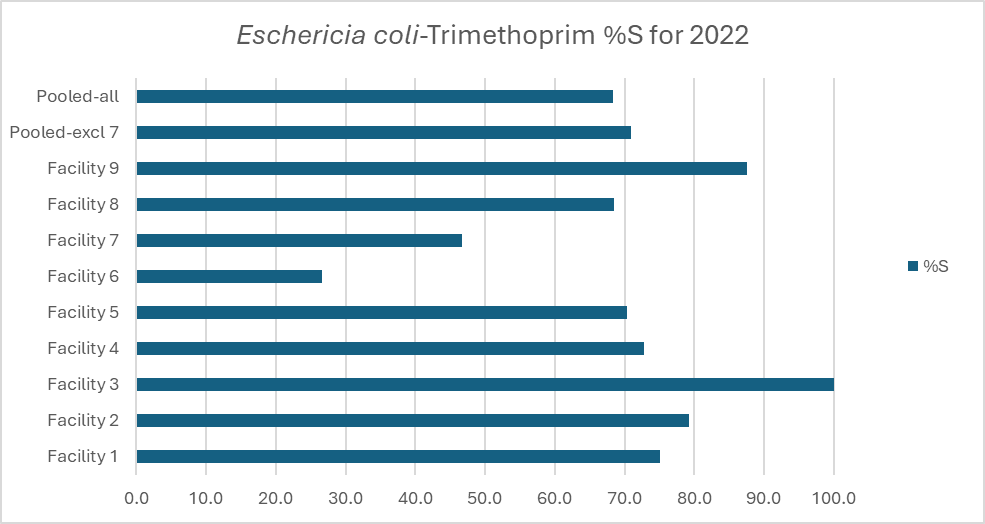


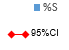


Percent susceptibility for Escherichia coli and Trimethoprim for 2022


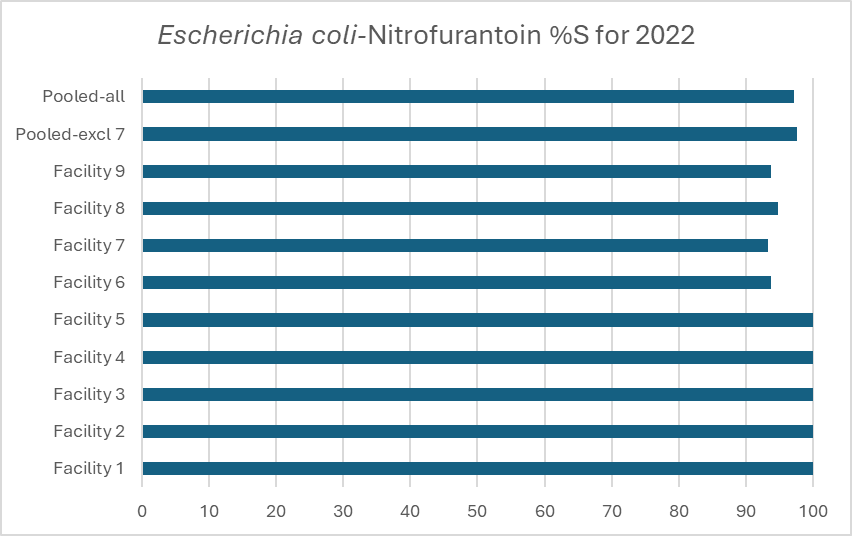


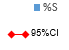


Percent susceptibility for Escherichia coli and Nitrofurantoin for 2022
