# Supplementary material for: Antibiogram development for Australian residential aged care facilities
Source: Infect Control Hosp Epidemiol. 2024 Sep 26;45(11):1325–31. doi: 10.1017/ice.2024.120 (PMC11663465; doi:10.1017/ice.2024.120)
Supplement: Khatri et al. supplementary material 4 — Khatri et al. supplementary material [file S0899823X2400120Xsup004.docx]

# Supplementary 4

Comparisons amongst different antibiograms using statistical analyses

**Facility 2- Biennial antibiogram data**

| **Pathogen-antibiotic Pairs** | | **Biennial (24 months)**  % S (n=total tested) | | |
| --- | --- | --- | --- | --- |
|  |  | %S (n) First isolate only | %%S (n) First isolate/12-months | P-value |
| *Escherichia coli* | Amoxicillin | 44.7(38) | 51.1(47) | 0.6636 |
|  | Amoxicillin/Clavulanate | 84.2(38) | 85.1(47) | 1 |
|  | Cefalexin | 92.1(38) | 93.6(47) | 1 |
|  | Nitrofurantoin | 97.4(38) | 97.9(47) | 1 |
|  | Trimethoprim | 73.7(38) | 78.7(47) | 0.6158 |
|  | Trimethoprim/sulfamethoxazole | 50(6) | 57.1(7) | 1 |
|  | Ciprofloxacin | 100(5) | 100(5) | 1 |
| *Klebsiella Pneumoniae* | Amoxicillin/Clavulanate | 100(7) | 100(7) | 1 |
|  | Cefalexin | 100(7) | 100(7) | 1 |
|  | Nitrofurantoin | 57.1(7) | 57.1(7) | 1 |
|  | Trimethoprim | 71.4(7) | 71.4(7) | 1 |
|  | Trimethoprim/sulfamethoxazole | 100(1) | 100(1) | 1 |
|  | Ciprofloxacin | 100(1) | 100(1) | 1 |
| *Enterococcus faecalis* | Ampicillin | 100(5) | 100(5) | 1 |
|  | Amoxicillin | 100(5) | 100(5) | 1 |
|  | Nitrofurantoin | 100(5) | 100(5) | 1 |
| *Pseudomonas aeruginosa* (Urine) | Ciprofloxacin | 100(3) | 100(3) | 1 |
| *Pseudomonas aeruginosa* (Skin) | Ciprofloxacin | 100(2) | 100(2) | 1 |
| *Staphylococcus aureus* | Amoxicillin/Clavulanate | 82.4(17) | 77.8(18) | 1 |
|  | Penicillin | 8.3(24) | 8(25) | 1 |
|  | Flucloxacillin | 79.2(24) | 76(25) | 1 |
|  | Cefalexin | 79.2(24) | 76(25) | 1 |
|  | Trimethoprim/sulfamethoxazole | 100(24) | 100(25) | 1 |
|  | Tetracycline | 100(24) | 100(25) | 1 |
|  | Clindamycin | 75(24) | 72(25) | 1 |

**Facility 2 - Triennial antibiogram data**

| **Pathogen-antibiotic Pairs** | | **Triennial (36 months)**  % S (n=total tested) | | |
| --- | --- | --- | --- | --- |
|  |  | %S (n) First isolate only | %%S (n) First isolate/12-months | P-value |
| *Escherichia coli* | Amoxicillin | 45.8(48) | 49.2(65) | 0.8491 |
|  | Amoxicillin/Clavulanate | 83.3(48) | 83.1(65) | 1 |
|  | Cefalexin | 89.4(47) | 92.2(64) | 0.7405 |
|  | Nitrofurantoin | 97.9(48) | 96.9(65) | 1 |
|  | Trimethoprim | 72.9(48) | 78.5(65) | 0.5116 |
|  | Trimethoprim/sulfamethoxazole | 50(10) | 57.1(14) | 1 |
|  | Ciprofloxacin | 83.3(6) | 88.9(9) | 1 |
| *Klebsiella Pneumoniae* | Amoxicillin/Clavulanate | 90.9(11) | 91.7(12) | 1 |
|  | Cefalexin | 90.9(11) | 91.7(12) | 1 |
|  | Nitrofurantoin | 63.6(11) | 58.3(12) | 1 |
|  | Trimethoprim | 81.8(11) | 75(12) | 1 |
|  | Trimethoprim/sulfamethoxazole | 50(2) | 66.7(3) | 1 |
|  | Ciprofloxacin | 50(2) | 66.7(3) | 1 |
| *Enterococcus faecalis* | Ampicillin | 100(10) | 100(10) | 1 |
|  | Amoxicillin | 100(10) | 100(10) | 1 |
|  | Nitrofurantoin | 100(10) | 100(10) | 1 |
| *Pseudomonas aeruginosa* (Urine) | Ciprofloxacin | 100(4) | 100(4) | 1 |
| *Pseudomonas aeruginosa* (Skin) | Ciprofloxacin | 100(5) | 100(5) | 1 |
| *Staphylococcus aureus* | Amoxicillin/Clavulanate | 80(15) | 80(15) | 1 |
|  | Penicillin | 2.9(35) | 2.9(35) | 1 |
|  | Flucloxacillin | 80(35) | 80(35) | 1 |
|  | Cefalexin | 79.4(34) | 79.4(34) | 1 |
|  | Trimethoprim/sulfamethoxazole | 100(35) | 100(35) | 1 |
|  | Tetracycline | 100(35) | 100(35) | 1 |
|  | Clindamycin | 77.1(35) | 77.1(35) | 0.793 |

**Facility 7- Biennial antibiogram data**

| **Pathogen-antibiotic Pairs** | | **Biennial (36 months)**  % S (n=total tested) | | |
| --- | --- | --- | --- | --- |
|  |  | %S (n) First isolate only | %%S (n) First isolate/12-months | P-value |
| *Escherichia coli* | Amoxicillin | 53.8(26) | 45.2(31) | 0.599 |
|  | Amoxicillin/Clavulanate | 88.5(26) | 90.3(31) | 1 |
|  | Cefalexin | 84.6(26) | 87.1(31) | 1 |
|  | Nitrofurantoin | 96.2(26) | 93.5(31) | 1 |
|  | Trimethoprim | 76.9(26) | 64.5(31) | 0.389 |
|  | Trimethoprim/sulfamethoxazole | 66.7(6) | 66.7(6) | 1 |
|  | Ciprofloxacin | 100(6) | 100(6) | 1 |
| *Klebsiella Pneumoniae* | Amoxicillin/Clavulanate | 100(7) | 100(8) | 1 |
|  | Cefalexin | 85.7(7) | 75(8) | 1 |
|  | Nitrofurantoin | 57.1(7) | 62.5(8) | 1 |
|  | Trimethoprim | 71.4(7) | 62.5(8) | 1 |
|  | Trimethoprim/sulfamethoxazole | 66.7(3) | 50(4) | 1 |
|  | Ciprofloxacin | 66.7(3) | 50(4) | 1 |
| *Enterococcus faecalis* | Ampicillin | 100(8) | 100(9) | 1 |
|  | Amoxicillin | 100(8) | 100(9) | 1 |
|  | Nitrofurantoin | 100(8) | 100(9) | 1 |
| *Pseudomonas aeruginosa* (Urine) | Ciprofloxacin | 100(3) | 100(3) | 1 |
| *Pseudomonas aeruginosa* (Skin) | Ciprofloxacin | 100(8) | 100(8) | 1 |
| *Staphylococcus aureus* | Amoxicillin/Clavulanate | 90.9(11) | 93.3(15) | 1 |
|  | Penicillin | 0(22) | 0(26) | 1 |
|  | Flucloxacillin | 90.9(22) | 92.3(26) | 1 |
|  | Cefalexin | 90.9(22) | 92.3(26) | 1 |
|  | Trimethoprim/sulfamethoxazole | 100(22) | 100(26) | 1 |
|  | Tetracycline | 100(22) | 100(26) | 1 |
|  | Clindamycin | 81.8(22) | 80.8(26) | 1 |

**Facility 7 - Triennial antibiogram data**

| **Pathogen-antibiotic Pairs** | | **Triennial (36 months)**  % S (n=total tested) | | |
| --- | --- | --- | --- | --- |
|  |  | %S (n) First isolate only | %%S (n) First isolate/12-months | P-value |
| *Escherichia coli* | Amoxicillin | 52.6(38) | 50(48) | 0.8313 |
|  | Amoxicillin/Clavulanate | 92.1(38) | 93.8(48) | 1 |
|  | Cefalexin | 89.5(38) | 91.7(48) | 0.7278 |
|  | Nitrofurantoin | 97.4(38) | 95.8(48) | 1 |
|  | Trimethoprim | 73.7(38) | 68.8(48) | 0.6416 |
|  | Trimethoprim/sulfamethoxazole | 71.4(7) | 75(8) | 1 |
|  | Ciprofloxacin | 100(7) | 100(8) | 1 |
| *Klebsiella Pneumoniae* | Amoxicillin/Clavulanate | 90.9(11) | 92.9(14) | 1 |
|  | Cefalexin | 90.9(11) | 78.6(14) | 0.6043 |
|  | Nitrofurantoin | 72.7(11) | 71.4(14) | 1 |
|  | Trimethoprim | 90.9(11) | 71.4(14) | 0.3406 |
|  | Trimethoprim/sulfamethoxazole | 75(4) | 50(6) | 0.5714 |
|  | Ciprofloxacin | 100(3) | 60(5) | 0.4643 |
| *Enterococcus faecalis* | Ampicillin | 100(10) | 100(12) | 1 |
|  | Amoxicillin | 100(10) | 100(12) | 1 |
|  | Nitrofurantoin | 100(10) | 100(12) | 1 |
| *Pseudomonas aeruginosa* (Urine) | Ciprofloxacin | 100(5) | 100(6) | 1 |
| *Pseudomonas aeruginosa* (Skin) | Ciprofloxacin | 91.7(12) | 92.3(13) | 1 |
| *Staphylococcus aureus* | Amoxicillin/Clavulanate | 87.5(8) | 93.3(15) | 1 |
|  | Penicillin | 3.4(29) | 2.4(41) | 1 |
|  | Flucloxacillin | 89.7(29) | 92.7(41) | 0.6864 |
|  | Cefalexin | 89.7(29) | 92.7(41) | 0.6864 |
|  | Trimethoprim/sulfamethoxazole | 100(29) | 100(41) | 1 |
|  | Tetracycline | 93.1(29) | 95.1(41) | 1 |
|  | Clindamycin | 82.8(29) | 82.9(41) | 1 |
