# Supplementary material for: Antibiogram development for Australian residential aged care facilities
Source: Infect Control Hosp Epidemiol. 2024 Sep 26;45(11):1325–31. doi: 10.1017/ice.2024.120 (PMC11663465; doi:10.1017/ice.2024.120)
Supplement: Khatri et al. supplementary material 5 — Khatri et al. supplementary material [file S0899823X2400120Xsup005.docx]

# Supplementary 5

Comparison of selected pathogen-antibiotic pairs for pooled antibiogram data for 2020, 2021 and 2022.

| **Pathogen-antibiotic Pairs** | | **Pooled RACF data**  % S (n=total tested) | | | |
| --- | --- | --- | --- | --- | --- |
|  |  | 2020 | 2021 | 2022 | P-value |
| *Escherichia coli* | Amoxicillin | 48.1(106) | 50(116) | 57.9(126) | 0.2659 |
|  | Amoxicillin/Clavulanate | 86.8(106) | 88.8(116) | 86.5(126) | 0.8436 |
|  | Cefalexin | 85.6(104) | 87.6(113) | 86.5(126) | 0.8837 |
|  | Nitrofurantoin | 97.2(106) | 97.4(117) | 97.6(125) | 1 |
|  | Trimethoprim | 68.9(106) | 70.1(117) | 71(124) | 0.9343 |
|  | Trimethoprim/sulfamethoxazole | 59.5(37) | 54.2(24) | 59.3(27) | 0.9254 |
|  | Ciprofloxacin | 41.7(24) | 57.9(19) | 58.3(12) | 0.5364 |
| *Klebsiella Pneumoniae* | Amoxicillin/Clavulanate | 96.6(29) | 100(18) | 100(24) | 1 |
|  | Cefalexin | 96.6(29) | 100(18) | 95.8(24) | 1 |
|  | Nitrofurantoin | 60(30) | 68.4(19) | 52.4(21) | 0.6124 |
|  | Trimethoprim | 80(30) | 84.2(19) | 91.7(24) | 0.5013 |
|  | Trimethoprim/sulfamethoxazole | 70(10) | 100(3) | 80(5) | 0.7794 |
|  | Ciprofloxacin | 50(6) | 75(4) | 100(1) | 0.7273 |
| *Enterococcus faecalis* | Ampicillin | 100(23) | 100(9) | 92.9(14) | 0.5 |
|  | Amoxicillin | 100(23) | 100(9) | 92.9(14) | 0.5 |
|  | Nitrofurantoin | 100(23) | 100(9) | 92.9(14) | 0.5 |
| *Pseudomonas aeruginosa* (Urine) | Ciprofloxacin | 100(12) | 90.9(11) | 81.8(11) | 0.2941 |
| *Pseudomonas aeruginosa* (Skin) | Ciprofloxacin | 100(14) | 80(5) | 75(8) | 0.1456 |
| *Staphylococcus aureus* | Amoxicillin/Clavulanate | 25(4) | 71.9(32) | 72.6(73) | 0.1533 |
|  | Penicillin | 10.4(67) | 12.9(62) | 15.6(64) | 0.715 |
|  | Flucloxacillin | 77.1(70) | 75.8(66) | 73.6(72) | 0.8994 |
|  | Cefalexin | 79.7(64) | 75(60) | 73(63) | 0.6992 |
|  | Trimethoprim/sulfamethoxazole | 94.2(69) | 96.9(65) | 100(73) | 0.1012 |
|  | Tetracycline | 92.9(70) | 98.5(66) | 98.6(73) | 0.1814 |
|  | Clindamycin | 79.7(69) | 81.8(66) | 93.2(73) | 0.0421 |
